# Acting Upon Uncertain Beliefs

**DOI:** 10.1007/s12136-019-00403-2

**Published:** 2019-08-24

**Authors:** Miloud Belkoniene, Patryk Dziurosz-Serafinowicz

**Affiliations:** 1 London’s Global University, London, UK; 2 University of Gdansk, Gdansk, Poland

**Keywords:** Decision-theory, Higher-order decision, Belief, Acceptance, Pragmatic encroachment on knowledge

## Abstract

This paper discusses the conditions under which an agent is rationally permitted to leave some uncertain propositions relevant to her decision out of her deliberation. By relying on the view that belief involves a defeasible disposition to treat a proposition as true in one’s reasoning, we examine the conditions under which such a disposition can be overridden and under which an agent should take into account her uncertainty as to a proposition she believes in the course of a particular deliberation. We argue that, in some contexts, an agent can be faced with the choice of either accepting or not accepting a proposition she believes in the course of her deliberation. We provide a description of such higher-order deliberations within the framework of expected utility theory and draw conclusions regarding the phenomenon of pragmatic encroachment on knowledge.

## Introduction

Expected utility theory recommends a course of action which maximizes subjective expected utility. In order to apply the decision rule prescribed by this theory, the agent needs to settle for a specification of her decision-problem. More precisely, she needs to specify the alternative options, a number of possible states-of-affairs which those options’ outcomes depend on, and a set of possible outcomes. This paper focuses on the way agents should specify the decision-problems they face by asking under what conditions an agent is rationally permitted to leave some uncertain propositions relevant to her decision out of her deliberation. This question is particularly pressing because expected utility theory provides an account of rational decisions taken under uncertainty. But for any decision-problem faced by a particular agent, there are numerous uncertain propositions relevant to her decision that could be taken into account in her deliberation and this raises the question of whether, and in what circumstances, an agent is rationally permitted to leave some of those possibilities out of her expected utility calculus.

According to one view recently put forward by Ross and Schroeder (2014), outright belief involves a defeasible disposition to treat its content as true in one’s reasoning even though one is not absolutely certain of its content. In Section 2, we present this account and show why it should be preferred to other accounts of the type of reasoning dispositions involved in outright belief. We then introduce Savage’s distinction between *small-world* and *grand-world* decisions and Joyce’s (1999) view of the commitment involved in small-world decisions to clarify in what sense the believer’s disposition to treat *p* as true in her reasoning is defeasible. In Section 3, we argue that an agent’s disposition to treat *p* as true in her reasoning can be overridden, in a particular choice situation, by her confidence that taking her uncertainty as to *p*’s truth into account in her deliberation would change her preferences. In addition, we argue that in case her disposition to treat *p* as true is overridden, the agent is faced with the choice of either accepting *p* or not accepting it in the context she is in. In doing so, we provide reasons to reject the Justification principle accepted by Ross and Schroeder and claim that an agent can be justified in occurrently believing that *p* although she should decide not to accept *p* in the course of a particular deliberation. In Section 4, we clarify the conditions under which an agent should accept a proposition that she believes in the course of a particular deliberation by providing a detailed description of the kind of higher-order choices that an agent faces when she has to determine whether to accept a given proposition. Finally, in Section 5, we rely on the clarifications provided in Section 4 to offer an alternative explanation of the phenomenon of pragmatic encroachment on knowledge which shows how knowledge’s sensitivity to the choice situation an agent is in depends on the agent’s degree of uncertainty as to what she believes.

## Beliefs and Rational Decisions

Expected utility theory takes rational preferences to be representable in terms of an agent’s rational expectations and her utility assignments to the possible outcomes of her options. When conjoined with probabilism, which states that rational expectations, or *credences*, are themselves representable as a probability distribution over a set of propositions, expected utility theory amounts to the view that rational preferences are representable in terms of probabilistically coherent expectations and utility assignments.^1^

To see how rational preferences can be represented according to expected utility theory, consider the following toy example: an agent has to choose between taking her umbrella before leaving her house and not taking her umbrella before leaving her house. We stipulate that only two possible states-of-affairs that are not under this agent’s control are relevant to specify the possible outcomes of these options; the first one being ‘it will rain’ and the second one being ‘it will not rain’. The possible outcomes of this agent’s options are the consequences that each option would have if either one of these states-of-affairs obtained. The decision-problem this agent faces can, accordingly, be represented by Table 1 where her options figure in the first column of the matrix, the states-of-affairs which these options’ possible outcomes depend on figure in the first row of the matrix and the possible outcomes figure in the remaining cells of the matrix.

**Table 1:** A simple decision-problem

	Rain	No rain
Take umbrella	Carry umbrella; do not get wet	Carry umbrella; do not get wet
Do not take umbrella	Do not carry umbrella; get wet	Do not carry umbrella; do not get wet

Expected utility theory prescribes that the agent faced with this decision-problem chooses the course of action which maximizes the utility that she can expect from her options, given the subjective probability that she assigns to the propositions in the first row of the matrix and the utility she assigns to the possible outcomes represented in the middle cells of the matrix. When a simple and idealized decision-problem such as the one represented in Table 1 is considered, expected utility theory allows a straightforward determination of which course of action should be preferred given an agent’s overall expectations and the utility she assigns to the possible outcomes of her options. However, it is important to acknowledge that decision-problems such as the one represented in Table 1 are *idealized* models of real-world decisions. In real-world situations, the decision-problems we face are far more complex than the one represented in Table 1. For instance, an agent who has to decide whether to take her umbrella before leaving her house is not normally certain that the proposition (*p*_1_): ‘my umbrella is not defective’ is true. She is not normally in a position to rule out the possibility of her umbrella being defective. But *p*_1_ is surely relevant to her decision and it might be rational for her to take her uncertainty about this proposition into account. In fact, for each decision-problem we face, we can always find many uncertain propositions relevant to our decision that are treated as true in the course of our deliberation: a proposition *p* being treated as true in the context of a deliberation when an agent evaluates her options conditional on its truth.^2^ It is therefore important to account for this aspect of practical deliberation.

One plausible explanation of the fact that we treat uncertain propositions as true in the course of our deliberation is that we believe them to be true although we are not absolutely certain that they are—i.e. our degree of credence in their truth is not maximal. Contrary to the notion of credence which corresponds to an agent’s degree of confidence in the truth of a given proposition, the notion of belief corresponds to a binary or on-off attitude which is not obviously reducible to the degreed attitude of credence.^3^ In addition, while belief consists of a binary attitude adopted toward the truth of a given proposition, it does not necessarily involve being certain of the truth of its content. One can believe that *p* and rationally do so without being absolutely certain that *p* is true. These aspects of belief explain why this attitude can help to account for the fact that some uncertain propositions that are relevant to the choice situations we face are treated as true in our deliberations.

This explanation suggests that the attitude of belief involves a disposition to treat a proposition as true in one’s reasoning. But, as belief does not necessarily involve certainty, it might not always be rationally permissible for an agent to treat a proposition that she believes as true in the course of a particular deliberation. As a matter of fact, we do not always act upon our beliefs and if belief involves a disposition to treat a proposition as true in one’s reasoning, it is necessary to explain why, in some contexts, agents do not treat what they believe as true in the course of their deliberations.

According to Ross and Schroeder (2014), the disposition involved in belief is a *defeasible* disposition that might be overridden in some contexts. They (2014, pp. 267–268) offer the following account which they label the *Reasoning Disposition Account of Belief*:

**Reasoning Disposition Account:** To believe that *p* is, partly, to be defeasibly disposed to treat *p* as true in one’s reasoning whenever *p* is relevant to the decision one’s faces.^4^

This account provides an explanation of why agents do not always treat what they believe as true in the course of their deliberations. Although an agent’s belief that *p* disposes her to treat *p* as true in her reasoning, this disposition might be overridden in a particular context. Other philosophers, instead of conceiving the kind of disposition involved in belief as being defeasible, take it to be relative to certain choice situations. According to Weatherson:

To believe that *p*, there must be some decision-problem such that some table the agent would be disposed to use to solve it encodes that *p*. If there is no such problem, the agent does not believe that *p*. (Weatherson 2016, p. 229)

Likewise, Locke (2014) relativizes the disposition involved in belief to a certain class of choice situations. According to him (2014, p. 33), belief involves an *indefeasible* disposition to treat *p* as true in at least some choice situations instead of involving a *defeasible* disposition to treat *p* as true whenever *p* is relevant to a choice situation.

This competing account of the disposition involved in belief can also explain why agents do not always act on what they believe. Yet, we see at least two problems with it. First, the explanation provided by Locke’s account raises several questions that are not addressed by Locke. Suppose that an agent who believes that *p* does not treat *p* as true in her reasoning in the choice situation *C* in spite of the fact that *p* is relevant to her decision. According to Locke, what explains this is that the agent, while she believes that *p*, is not disposed to treat *p* as true in this particular choice situation. But this explanation raises another fundamental question: what makes it that in some choice situations the believer is disposed to treat *p* as true, while she is not disposed to do so in situation *C*? Locke (2014, p. 44) admits, for instance, the possibility of two agents having a belief concerning the same proposition and being disposed to treat the believed proposition as true in different choice situations. But then one may ask: do these two agents have different beliefs and, if so, why are they different while having the same propositional content? The account of the reasoning disposition involved in belief offered by Locke thus raises several questions regarding the nature of belief. In particular, the assumption Locke relies on to explain why an agent who believes that *p* is not disposed to treat a proposition *p* as true in a particular choice situation is in need of further explanation and unless Locke provides this explanation, Ross and Schroeder’s Reasoning Disposition Account constitutes a better explanation of this phenomenon. The Reasoning Disposition Account does not raise the type of questions raised by Locke’s account, for it says that believing that *p* involves a defeasible disposition to treat *p* as true *wheneverpis relevant to one’s decision*. On this account, there is no question of why the agent’s belief disposes her to treat a proposition as true in some choice situations and not in other choice situations.

The second problem with Locke’s account is that if believing that *p* only involves being disposed to treat *p* as true in some choice situations, it is, in principle, possible for an agent to believe that *p* and to be disposed to treat *p* as true in only some very remote choice situations. But in what sense does an agent who is disposed to treat *p* as true in almost no choice situations still count as believing that *p*? One could try to argue that if an agent is disposed to treat *p* as true in sufficiently many choice situations, then this agent counts as believing that *p*. Yet, this directly raises the question as to how the threshold should be determined. Is there a non-arbitrary way to fix it? The contextual route is not an option in that case, as we are talking about an agent’s disposition to treat a proposition as true in some choice situations.

The above considerations give us reasons to prefer the Reasoning Disposition Account as an explanation of the fact that agents do not always treat what they believe as true in their reasoning. In the next sections of the present paper, we will rely on this account to elucidate the conditions under which it is rationally permissible for an agent to act upon her beliefs and to treat what she believes as true in the context of a particular deliberation. For now, let us introduce an idea put forward by Joyce (1999, Ch. 2.6) concerning what Savage (1954, Ch. 5) called *small-world decisions* that can allow clarifying in what sense the kind of disposition involved in belief is defeasible. A small-world decision, as opposed to a grand-world decision, is a decision in which only a subset of all the courses of action one could choose at a given time and only a subset of all the uncertain propositions relevant to specify the possible outcomes of those options are considered. It should be clear that agents with limited cognitive capacities are bound to take small-world decisions. Grand-world decisions are far too complex to be handled. Now, according to Joyce (1999), any small-world decision involves a tacit commitment on the part of the agent to the view that her preferences would not be different if more options and more uncertain propositions relevant to specify the possible outcomes of those options were taken into account. He adds that:

A rational small-world decision maker should always make choices in a state in which she feels confident that her evaluations of the relative merits of acts would not be overturned if she were to reflect on her predicament more fully. (Joyce 1999, p. 76)

To see why it is plausible that small-world decisions involve this kind of commitment, consider again the decision-problem represented in Table 1. If the agent facing this decision-problem was very confident that taking into account her uncertainty about *p*_1_ would change her preferences, it seems clear that simply deliberating according to Table 1 would be problematic. In such a case, she should take her uncertainty about this proposition into account, even though she believes that this proposition is true.

## Accepting Believed Propositions

Let us consider the following case, an adapted version of a case from Ross and Schroeder (2014, p. 261), to see how the kind of commitment which, according to Joyce, is involved in small-world decisions can help clarify the sense in which the reasoning disposition involved in belief is defeasible:

*Peanut Allergy*: Sarah made three sandwiches—one containing tuna, one containing almond butter and one containing peanut butter—that she placed in the fridge. The sandwich containing tuna was placed on the left, the one containing peanut butter in the middle and the one containing almond butter on the right. Sarah’s nephew Algernon is visiting for lunch, and Sarah is aware that he has a severe peanut allergy. Sarah also believes that the sandwich containing peanut butter is placed in the middle of the fridge and her credence for this is .8 (the sandwiches were placed in the fridge hours ago and Sarah is well aware that her memory is not infallible). In addition, while Sarah believes that Algernon likes almond butter, she is just slightly more confident that he does not like tuna than the contrary (her credence that he does not like tuna is .55). Algernon asks Sarah for a sandwich. When Sarah goes to the fridge, she can tell, by visual inspection, which is the tuna sandwich, but she cannot tell which is the peanut butter sandwich and which is the almond butter sandwich.

Sarah believes that the sandwich placed on the right does not contain peanut. By the Reasoning Disposition Account, this means that she is defeasibly disposed to treat the proposition (*p*_2_): ‘the sandwich placed on the right does not contain peanut’ as true in the course of her deliberation. The way Sarah is disposed to deliberate in order to determine what she prefers to do when her nephew asks her for a sandwich can thus be represented by Table 2, where ‘the almond butter sandwich’ is, given what Sarah believes, the one placed on the right.

**Table 2:** A high-stake decision-problem

	Like tuna	Do not like tuna
Give tuna sandwich	Algernon likes the sandwich	Algernon does not like the sandwich
Give almond butter sandwich	Algernon likes the sandwich	Algernon likes the sandwich

As Sarah is willing to please her nephew and is more confident that he does not like tuna than the contrary, if she deliberated according to Table 2, she should decide to give Algernon the sandwich that is placed on the right in the fridge. The expected utility of this option would be the highest. However, given what is at stake in this case and given that Sarah is not absolutely certain that the sandwich placed on the right does not contain peanut, it seems that Sarah should not deliberate according to Table 2. More specifically, it seems that she should not treat *p*_2_ as true in the context of her deliberation, although she believes that *p*_2_ is true.

The kind of commitment that is involved in small-world decisions according to Joyce can help to account more precisely for the situation described in the Peanut Allergy case. Although Sarah believes that *p*_2_ is true, assuming that she is aware of what is at stake—her nephew’s life—there is a risk that taking her uncertainty as to *p*_2_ into account would change her preferences. That is, given her degree of uncertainty as to *p*_2_ and what is at stake, there is a risk that deliberating according to Table 2 would not result in the same decision as the decision she would take if she deliberated according to Table 3.

**Table 3:** A high-stake decision-problem (extended)

	Like tuna;	Like tuna;	Do not like tuna;	Do not like tuna;
	peanuts	no peanuts	peanuts	no peanuts
Give tuna sandwich	Algernon likes the sandwich	Algernon likes the sandwich	Algernon does not like the sandwich	Algernon does not like the sandwich
Give sandwich on the right	Algernon has an allergic shock	Algernon likes the sandwich	Algernon has an allergic shock	Algernon likes the sandwich

We suggest that Sarah’s confidence that taking her uncertainty as to *p*_2_’s truth would change her preferences is precisely what can override, if sufficiently high, her disposition to treat *p*_2_ as true in her reasoning. In other words, her belief that *p*_2_ defeasibly disposes her to treat *p*_2_ as true in her reasoning, in the sense that she is disposed to reason in such a way insofar as her confidence that taking into account her uncertainty as to *p*_2_’s truth in the course of her deliberation would change her preferences is not higher than a given threshold.^5^ In addition, in case Sarah’s disposition is overridden, she is presented with a choice: either taking her uncertainty as to *p*_2_’s truth into account in her deliberation or not taking it into account. This is so because what overrides Sarah’s disposition to treat *p*_2_ as true in her reasoning is her confidence—but not her absolute certainty—that her preferences would not be the same if her uncertainty as to *p*_2_’s truth was taken into account. A context in which an agent’s disposition to treat *p* as true in her reasoning is overridden thus corresponds to a specific choice situation. In such a context, the agent has a choice between taking the risk of leaving some information critical to her decision out of her deliberation—that is, the possibility that ¬*p*—or taking this information into account in spite of her belief that *p* is true. In the next section, we will discuss in more details the factors that are relevant to determine an agent’s confidence that her preferences would be overturned if her uncertainty as to *p*’s truth was taken into account in the course of her deliberation. For now, let us focus on another question raised by this proposal, which concerns the justificatory status of the agent’s belief in contexts in which it is not rational for the agent to treat the believed proposition as true in the course of her deliberation.^6^

According to Ross and Schroeder (2014, p. 271), an agent is not justified in occurrently believing that *p* in contexts in which it is not rational for her to treat *p* as true in the course of a deliberation, although she believes dispositionally that *p*. They (2014, p. 271) rely on the following principle to motivate this claim:

**Justification principle:** If having attitude *a* essentially involves being disposed to *ϕ* under circumstance *C*, then an agent *A* is justified to occurrently have attitude *a* in *C* only if it is rationally permissible for *A* to *ϕ* in *C*.

It follows straightforwardly from the Justification principle and the Reasoning Disposition Account that if an agent believes that *p* and is not rationally permitted to treat *p* as true in a given choice situation, then she is not justified, in that situation, to occurrently believe that *p*. One advantage of the conjunction of these principles is that it allows offering an explanation of the phenomenon of pragmatic encroachment on knowledge. However, as outlined by Locke (2014), the conjunction of the Justification principle and the Reasoning Disposition Account also leads to seriously counterintuitive consequences. Locke considers the following case:

*Liz’s Bet:* One day Liz is offered a bet on whether she was born in England. In fact, Liz was born in England, and her reasons for believing so are just like anyone else’s: her parents told her she was born in England, her aunts and uncles tell stories about going to see her in the hospital, she has never had a problem when dealing with the government, and so on. However, the terms of the bet are as follows: if Liz was born in England, Liz gains *£*1; if Liz was not born in England, Liz is tortured for the next 30 years. (Locke 2014, p. 39)

As pointed out by Locke, given the evidence Liz has in this case, it is very hard to deny that she is justified in occurrently believing that she was born in England—that is, in consciously believing that she was born in England in this particular context. On the other hand, given the bet that is offered to her, it seems that she should not act on her belief and that she should not treat the proposition ‘I was born in England’ as true when deciding whether or not to take the bet. According to Locke, the Reasoning Disposition Account goes wrong in requiring that Liz has any disposition to treat the proposition ‘I was born in England’ as true in this situation. By his own account, it is possible for Liz to believe this proposition and yet not to be disposed to treat it as true in this particular choice situation. But, as outlined in the previous section, there are serious reasons to be dissatisfied with Locke’s analysis of Liz’s Bet case.

We believe that the problem raised by Liz’s Bet case results rather from the Justification principle than from the Reasoning Disposition Account. Let us first note that the Justification principle is not as intuitive as it seems to be when we consider more carefully the distinction that can be made between dispositional belief and occurrent belief. Ross and Schroeder (2014, p. 272) note that given this principle, an agent can be justified in having the dispositional attitude *a* in *C* without being justified in occurrently having this attitude in *C*. But this situation appears somewhat odd. For when the reasons one has for having (dispositionally) the belief that *p* justify having this belief in *C*, why are they not also sufficient to justify occurrently believing that *p*? In addition to the Justification principle, a principled explanation of why a subject needs more reasons for being justified in having the occurrent belief that *p* than for being justified in having the dispositional belief that *p* is required.

Setting aside the question of the initial plausibility of the Justification principle, let us now focus on what we take to be the main problem of this principle. According to the Justification principle, the fact that it is not rationally permissible for an agent to treat *p* as true in the course of her deliberation means that the agent, in the choice situation she is in, lacks justification for occurrently believing that *p*. But the fact that it is not rationally permissible for an agent to treat *p* as true in the course of a deliberation can actually mean many different things. It could mean, for instance, that the agent should not *suppose* that *p* in the course of her deliberation. When one supposes that *p*, one treats *p* as true in the course of a deliberation. It could also mean that the agent should not *acceptp* in the course of her deliberation. Now, the fact that an agent should not suppose that *p* in a given context does not necessarily mean that she lacks justification for occurrently believing *p* in that context. This point is even clearer in the case of acceptance which is more closely related to the way one acts than the case of supposition. The attitude of acceptance is generally regarded as being under our direct voluntary control and as involving treating a proposition as true in the sense of being ready to act as if it was true. Consider the following definition from Cohen:^7^

To accept that *p* is to have or adopt a policy of deeming, positing, or postulating that *p*—i.e. of including that proposition or rule among one’s premises for deciding what to do or think in a particular context, whether or not one feels it to be true that *p*. (Cohen 1992, p.4)

Cohen’s definition makes it clear that (i) acceptance is an attitude adopted in the context of a particular deliberation, (ii) that this attitude consists in treating a given proposition as true in one’s reasoning, and, finally, (iii) that one can decide voluntarily to accept or not to accept a given proposition, regardless of what one believes (or feels to be true in Cohen’s terminology).

With this characterization in mind, it is plausible that the fact that an agent should not decide to accept a given proposition in the course of a particular deliberation does not necessarily mean that she lacks justification for occurrently believing this proposition. Take the case of a lawyer discussed by Cohen (1992, p. 20) who has every reason to believe that her client is guilty of the crime she is accused of. As she has to defend her client, it is rational for the lawyer to deliberate as if her client was not guilty. It is rational for her not to accept the proposition ‘my client is guilty’ when she deliberates about the case and makes decisions in court in spite of the reasons she has for believing it. But the fact that the lawyer should choose not to accept the proposition ‘my client is guilty’ does not mean that she lacks justification for believing it occurrently or dispositionally.

If it is not rationally permissible for an agent to treat *p* as true in the course of a deliberation in the sense that she should not accept *p* in this context, one cannot thereby conclude that she lacks justification for occurrently believing that *p*. It might be the case that, in the particular choice situation the agent is in, she should not accept that *p* despite her disposition to treat *p* as true in this choice situation and despite the justification she has for believing—both dispositionally and occurrently—that *p*. The Justification principle is problematic because it abstracts from the various reasons for which, in a particular choice situation, it may not be permissible for an agent to treat *p* as true. Yet, in Liz’s Bet case for instance, it is quite plausible that Liz should not treat the proposition ‘I was born in England’ as true in the sense that she should decide not to accept this proposition in spite of her disposition to treat it as true, and in spite of the justification she has for believing it. The fact that Liz should not treat this proposition as true in that sense does not mean that she lacks justification for occurrently believing it. As Locke puts it, if anyone has justification for believing anything, then Liz has justification for believing that she was born in England. What the Justification principle overlooks is the fact that although the attitude of belief essentially involves a disposition to treat *p* as true, the fact that it is not rationally permissible for an agent to treat *p* as true might be related to facts that have nothing to do with the justification the agent has for believing that *p*, either dispositionally or occurrently. It might be related to facts that constitute reasons to decide not to treat *p* as true in spite of the justification the agent has for believing that *p*.

## Decisions to Accept

If we are correct, the Justification principle instead of the Reasoning Disposition Account should be rejected in light of cases such as Liz’s Bet case. But as we analyzed this case in terms of the believed proposition that Liz is justified to accept, it is necessary to examine more precisely the conditions under which it is rational for an agent to accept a proposition that she believes in a given choice situation. As the attitude of acceptance is an attitude adopted toward the truth of a proposition as a result of a choice, we suggest that the conditions under which it is rational to accept a given proposition can be modeled within the framework of expected utility theory. As already mentioned, within this framework, a decision-problem can be represented by a set of options, a set of possible states-of-affairs which those options’ outcomes depend on and the set of possible outcomes. We can think of each option as a function *f* that takes as argument a state-of-affairs *p*_*i*_ and returns an outcome *f*(*p*_*i*_) that depends on whether *p*_*i*_ obtains. A decision-problem can thus be represented by a decision-matrix such as Table 4.

**Table 4:** An abstract decision-problem

	***p***_***1***_	***p***_***2***_	***…***
***f***	*f*(*p*_1_)	*f*(*p*_2_)	...
***g***	*g*(*p*_1_)	*g*(*p*_2_)	...
**...**	...	...	...

The kind of choice faced by an agent who has to decide whether to accept *p* can be conceived of as a higher-order decision-problem and represented in the same way. Such decision-problems are higher-order problems because they relate to a choice made concerning the way to deliberate about another, lower-order, decision-problem. When an agent deliberates about whether to accept *p* in a particular choice situation, she deliberates about whether to take her uncertainty about the truth of *p* into account in the course of her deliberation regarding another, lower-order, decision-problem. Those higher-order decision-problems can be represented in the same way as lower-order decision-problems because they also involve a set of options, a set of possible states-of-affairs relevant to determine the possible outcomes of those options and a set of possible outcomes. Let us specify each of these sets in turn.

We are concerned here with higher-order deliberations that result from an agent’s confidence that taking her uncertainty about a certain proposition into account in her lower-order deliberation would change her preferences regarding the lower-order decision-problem she faces. For this reason, the kind of higher-order decision-problems we are concerned with always involves two options: accepting *p* and not accepting *p*. Of course, one first-order decision-problem can give rise to more than one higher-order decision-problems. Given the same first-order decision-problem, an agent can be faced with the choice of whether to accept *p*_*i*_ and whether to accept *p*_*j*_, where *p*_*i*_ and *p*_*j*_ are not equivalent. The agent might indeed be confident that taking her uncertainty about *p*_*i*_ into account in her lower-order deliberation would change her preferences and might also be confident that taking her uncertainty about *p*_*j*_ into account would change her preferences. But each of these higher-order decision-problems involves exactly two options: accepting a certain proposition or not accepting it. This is because each of them results from the agent’s confidence that taking into account her uncertainty about a certain proposition—*p*_*i*_ or *p*_*j*_—would change her first-order preferences.

For their part, the possible states-of-affairs that are relevant to determine the possible outcomes of the agent’s options consist of *p* ∗ and ¬*p* ∗ where *p* ∗ stands for: ‘my (lower-order) decision depends on the fact that my uncertainty as to the truth of *p* is taken into account in my deliberation’. More generally, for any agent *A* facing a decision-problem *D*, *p* ∗ stands for the proposition: ‘my preferences concerning the options in *D* depend on the fact that my uncertainty as to the truth of *p* is taken into account in my deliberation’. Accordingly, for each higher-order decision-problem, there are exactly two possible states-of-affairs relevant to determine the possible outcomes of the agent’s options. Either the agent’s first-order preferences depend on the fact that her uncertainty about *p* is taken into account in the context of her first-order deliberation or they don’t. The reason is, here also, that each of those higher-order decision-problems is relative to the agent’s confidence that taking her uncertainty about the truth of a particular proposition into account would change her first-order preferences. The decision-problem an agent faces when she has to determine whether she should accept a certain proposition is thus always a grand-world decision-problem in which figure all the agent’s options and all the possible states-of-affairs relevant to specifying the possible outcomes of those options. An agent’s decision to accept or not to accept a proposition, therefore, does not involve the kind of commitment involved in small-world decision-problems.^8^

Before turning to the possible outcomes of accepting or not accepting a certain proposition in the course of a given deliberation, let us offer some clarifications concerning the agent’s credence in *p* ∗ which, if above a certain threshold, can override the agent’s disposition to treat *p* as true in the course of her deliberation. That credence, if rational, depends on two factors: the agent’s credence for ¬*p*—that is, $1-\Pr (p)$ where $\Pr (p)$ stands for the agent’s credence for *p*—and the utility that the agent would assign to the possible outcomes of her options if her uncertainty about *p* was taken into account in the context of her deliberation.

Consider Table 5. Sarah’s credence that taking her uncertainty as to *p*_2_—i.e. ‘the sandwich placed on the right does not contain peanut’—would change her preferences depends, if rational, on her credence for ¬*p*_2_ which is equivalent to the disjunction representing the possible states-of-affairs in dark grey. In addition, it depends on the utility she would assign to the possible outcomes that depend on ¬*p*_2_—i.e. the outcomes in light grey. This is because the risk that taking her uncertainty about *p*_2_ into account would change her preferences increases with the deviation of the utility she would assign to the outcomes that depend on ¬*p*_2_ from the utility she would assign to the outcomes that depend on *p*_2_ and with the probabilities that weigh these utilities—i.e. the probability that Sarah assigns to ¬*p*_2_ and *p*_2_. The deviation of these utilities reflects, in fact, the stakes involved in the choice situation from the perspective of the agent. As Sarah knows about her nephew’s allergy and cares about his life, the utility she would assign to the possible outcomes that depend on ¬*p*_2_ varies greatly from the utility she would assign to the possible outcomes that depend on *p*_2_. A great negative utility would be assigned to ‘Algernon has an allergic shock’ while the utility assigned to the other possible outcomes would be much closer to 0. It is important to note, however, that Sarah does not need to deliberate about her decision-problem according to Table 5 in order to arrive at a certain confidence that taking her uncertainty about *p*_2_ into account would change her preferences. She can rely on her credence in *p*_2_ and in ¬*p*_2_ and on an estimate of the deviation of the utility she would assign to the outcomes that depend on ¬*p*_2_ from the utility she would assign to the outcomes that depend on *p*_2_—an estimate that corresponds to an estimate of the stakes related to *p*_2_’s truth in the choice situation she is in. To arrive at a certain credence in *p* ∗, an agent can thus rely on her credence in *p* and ¬*p* and on an estimate of the stakes related to *p*’s truth in the choice situation she is in. This credence should reflect the risk that taking her uncertainty for *p* into account would change her preferences which itself depends on these two factors.^9^^,^^10^

**Table 5:**
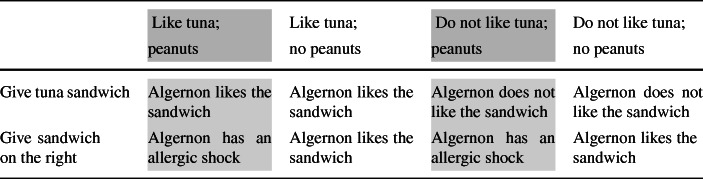
A high-stake decision-problem (extended)

Let us now turn to the possible outcomes of accepting or not accepting a certain proposition in the context of a particular deliberation. Given what has just been claimed concerning the set of options and the set of states-of-affairs relevant to specifying the outcomes of those options, there are two obvious outcomes that such decisions can lead to. If the agent’s first-order preferences depend on the fact that her uncertainty about *p* is taken into account in her first-order deliberation, then accepting *p* will result in a sub-optimal first-order decision and not accepting *p* will result in an optimal first-order decision. If they do not, accepting *p* as well as not accepting *p* will result in an optimal first-order decision. What we mean here by optimal and sub-optimal decisions can be clarified in terms of better or worse approximation to the first-order grand-world decision faced by an agent in a particular context. This grand-world decision is a hypothetical decision-problem in which all the information relevant to determining the agent’s preferences is taken into account. If an agent’s preferences depend on the fact that her uncertainty about *p* is taken into account in her deliberation and the agent nonetheless decides to accept *p*, her decision will be sub-optimal in the sense that some information, critical to her preferences, will not be taken into account in her deliberation. Her decision will be a worse approximation to the grand-world decision she faces than the decision in which her uncertainty about *p* is taken into account. In addition to the optimality or sub-optimality of her first-order decision, another possible outcome of a decision to accept or not to accept *p* in the course of a first order-deliberation is the possible additional cognitive cost related to the agent’s acceptance of *p*. As a matter of fact, deliberating according to Table 2 is less costly, cognitively speaking, than deliberating according to Table 5. This means that deciding not to accept *p*_2_ will result in a first-order decision which is cognitively more costly.^11^

The kind of deliberation in which an agent engages when she has to determine whether to accept a proposition in the context of a particular deliberation can now be captured in a decision-matrix of the same form as Table 4. Let *A* be an agent who faces a decision-problem *D* such that the states-of-affairs *p* and ¬*p* are relevant to specifying the possible outcomes in *D*. Let *p* ∗ stand for the proposition ‘my preferences concerning the options in *D* depend on the fact that my uncertainty as to the truth of *p* is taken into account in the context of my deliberation’. In case *A* has to choose between accepting *p* or not accepting *p* in the context of her lower-order deliberation, *A*’s higher-order deliberation can be captured in Table 6.

**Table 6:** A’s higher-order decision-problem

	***p∗***	***¬p∗***
Accept ***p***	Sub-optimal lower-order decision; no additional cognitive cost	Optimal lower-order decision; no additional cognitive cost
Do not accept ***p***	Optimal lower-order decision; additional cognitive cost	Optimal lower-order decision; additional cognitive cost

The rationality of accepting *p* depends, for any particular first-order deliberation, on the agent’s credence in *p* ∗, on the utility she assigns to taking an optimal or sub-optimal first-order decision and on the negative utility she assigns to the additional costs resulting from not accepting *p*. The utility assigned to those possible outcomes depends on the choice situation the agent is in. For instance, given what is at stake in the Peanut Allergy case, Sarah will assign a high utility to taking an optimal first-order decision. In contrast, in a low-stake choice situation, the utility assigned by the agent to taking an optimal first-order decision will be much lower.

## Uncertain Belief, Knowledge and Acceptability

In light of the proposed elucidation of the conditions under which an agent is permitted to accept a given proposition in a particular choice situation, let us come back to the Reasoning Disposition Account. According to our proposal, the conditions under which an agent is rationally permitted to accept a proposition that she believes can be captured in terms of higher-order decisions. In case an agent who believes that *p* should decide not to accept *p* in a particular choice situation, she is not rationally permitted to treat what she believes as true in her reasoning. But, we claimed, it does not follow from this that the agent is not justified in believing that *p* in that particular choice situation. The justification she has for believing that *p* is not dependent on the rationality of the type of higher-order decisions discussed in the previous section. While this proposal, in maintaining the Reasoning Disposition Account, manages to accommodate our intuitions in cases such as the Peanut Allergy case or Liz’s Bet case, it also raises an important question regarding knowledge.

As already mentioned, the conjunction of the Reasoning Disposition Account with the Justification principle allows Ross and Schroeder to explain the phenomenon of pragmatic encroachment on knowledge. This phenomenon is related to a plausible connection between knowledge and rational action captured by the following principle:

**Knowledge action principle:** For any agent *A* and proposition *p*, if *A* is in a choice situation in which *A* could not rationally act as if *p*, then *A* does not know that *p*. (Ross and Schroeder 2014, p.262)

Assuming that this principle holds, in the Peanut Allergy case, Sarah cannot count as knowing that *p*_2_ if it is not rational for her to treat *p*_2_ as true in her reasoning. Likewise, in Liz’s Bet case, Liz cannot count as knowing that she was born in England in case it is not rational for her to take the bet that is offered to her. The conjunction of the Reasoning Disposition Account with the Justification principle allows explaining this under the assumption that knowledge requires justification for occurrently believing that something is the case. As in the Peanut Allergy case and in Liz’s Bet case, it is not rationally permissible for the agent to treat what she believes as true in her reasoning, it follows from the Justification principle that the agent is not justified in occurrently believing what she is disposed to treat as true in her reasoning. Given the above assumption concerning the justification of occurent belief and knowledge, this explains why the agent does not know the proposition that she is not rationally permitted to treat as true.

The question is now whether the phenomenon of pragmatic encroachment on knowledge can still be explained upon rejecting the Justification principle and maintaining the Reasoning Disposition Account. If it can’t be explained, this would speak in favor of maintaining the Justification principle in case the Knowledge action principle is correct. Fortunately, this phenomenon can still be explained in light of the following principle:

**Knowledge acceptability principle:** For any agent *A* and proposition *p*, if *A* is in a choice situation in which *A* could not rationally accept *p*, then *A* does not know that *p*.

The Knowledge acceptability principle is rooted in the idea, which can be traced back to Descartes, that knowledge is related to an act of voluntary judgment or acceptance. An agent truly knows that *p* only if *p* is rationally acceptable in the situation she is in. Given the considerations that have been put forward concerning acts of acceptance, the Knowledge acceptability principle should appear reasonable to anyone who is willing to accept the Knowledge action principle. Indeed, the Knowledge action principle aims at capturing the tight connection between what someone knows and the way she is rationally permitted to act. As belief involves a *defeasible* disposition to treat a proposition as true in one’s reasoning, the fact that knowledge requires belief cannot account for this connection. One does not always act upon one’s beliefs. In addition, in light of what we argued, the justification one has for holding a belief is not sufficient either to account for this connection. This is because, in a given choice situation, an agent may not be rationally permitted to act upon what she is justified in believing. The notion of acceptance, on the other hand, allows accounting for the connection that the Knowledge action principle aims at capturing. The choice situations in which an agent is rationally permitted to act as if *p* was true are the situations in which *p* is acceptable for her in the sense that it is rational for her to choose to accept *p* in the course of her deliberation. Hence, in case what one knows is closely connected to the way one is rationally permitted to act, it is plausibly because what an agent knows depends on what is rationally acceptable for her in a given choice situation.

According to the Knowledge acceptability principle, what explains the phenomenon of pragmatic encroachment on knowledge is not the fact that the justification an agent has for occurrently believing a proposition is sensitive to the choice situation she is in. What explains this phenomenon is rather the fact that knowledge entails acceptability and that the conditions under which a proposition is rationally acceptable for an agent are sensitive to the choice situation she is in. In addition, the proposed analysis of higher-order deliberations shows that the sensitivity of the conditions under which a proposition is acceptable for an agent to the choice situation that agent is in depends on her credence for that proposition. We argued that an agent’s confidence that taking her uncertainty about *p* into account in the course of her deliberation would change her preferences, if rationally determined, depends on her credence in *p* and in ¬*p* and on the utility she would assign to the possible outcomes that depend on *p* and ¬*p*. Moreover, according to what we claimed, this degree of confidence, which, if rational, can be represented as a probability measure, in turn, weighs the utility the agent assigns to the possible outcomes of accepting or not accepting *p*. This entails that the sensitivity of the conditions under which a proposition believed by an agent is acceptable depends on the agent’s credence in the believed proposition. If the agent’s credence for *p* is close to certainty, the range of choice situations in which there is a risk that taking her uncertainty about *p* into account in the course of her deliberation would change her preferences is limited. This is because, if her credence in ¬*p* is close to 0, then the risk of her preferences being overturned if she were to take her uncertainty about *p* into account is less sensitive to the deviation of the utility she would assign to the outcomes that depend on *p* from the utility she would assign to the outcomes that depend on ¬*p*. In contrast, if her credence in *p* is far from certainty, her confidence that taking her uncertainty about *p* into account would change her preferences is highly dependent on the context she is in. As her credence for ¬*p* is far from 0, in case the utility she would assign to the outcomes that depend on *p* deviates from the utility she would assign to the outcomes that depend on ¬*p*, the risk of her preferences being overturned if she were to take her uncertainty about *p* into account is high.

By the Knowledge acceptability principle, it follows that the sensitivity of what an agent knows to the choice situation she is in is a function of her degree of uncertainty for the propositions that she is justified in believing. If an agent who is justified in believing the true proposition *p* is certain that *p*, then her knowledge that *p* is not sensitive to the choice situations she is in. In any choice situation, *p* is acceptable for that agent. On the other hand, if an agent who is justified in believing the true proposition *p* is uncertain as to *p*’s truth, then the sensitivity of her knowledge to the choice situation she is in is proportional to her degree of uncertainty as to *p*’s truth. This allows the explanation provided by the conjunction of the Reasoning Disposition Account with the Knowledge acceptability principle to meet the intuition that the degree of uncertainty of the propositions that one justifiably believes impacts the range of choice situations in which one’s justified beliefs constitute knowledge.

The Reasoning Disposition Account and the Knowledge acceptability principle thus allow explaining the phenomenon of pragmatic encroachment on knowledge. In addition, the explanation has the intuitive consequence that the extent to which the acceptability of what one justifiably believes is sensitive to pragmatic considerations is a function of one’s degree of uncertainty as to what one believes. It is, however, important to note that this explanation does not exclude any impact of the acceptability of what one believes on the justification one has for holding some beliefs. We assumed here, for the sake of simplicity, that the result of one’s decision not to accept *p* in the context of a particular deliberation is a lower-level deliberation in which ¬*p* is taken into account. However, another possible result of such a decision can be a re-opening of the inquiry concerning the truth of *p*. If an agent who believes that *p* decides, as a result of a higher-order deliberation, not to accept *p*, she can engage in a lower-level deliberation in which her uncertainty about *p* is taken into account. But she can also re-open the question of whether *p* is true and look for additional evidence which could allow raising her credence in *p*. In contexts in which additional evidence for *p* is easily accessible, a decision not to accept that *p* will plausibly lead to a search for additional evidence. In the Peanut Allergy case, for instance, the most natural result of the decision not to accept *p*_2_ is simply to look for additional evidence which can confirm or dis-confirm the proposition instead of deliberating according to Table 5. Belief justification is thus indirectly impacted by the acceptability of what one believes. An agent’s decision not to accept a given proposition can lead her to re-open the question of that proposition’s truth and look for additional evidence that can either rationalize raising her credence for that proposition or revising her belief toward it.

## Conclusion

In this paper, we relied on Joyce’s claim that small-world decisions involve a particular kind of commitment to elucidate the conditions under which an agent is rationally permitted to act upon her beliefs and leave some uncertain propositions out of her expected utility calculus. We argued that Ross and Schroeder are right in taking belief to involve a defeasible disposition to treat a proposition as true in one’s reasoning and that this disposition can be overridden by an agent’s confidence that taking into account her uncertainty as to the believed proposition’s truth in her deliberation would change her preferences. We then argued that the Justification principle accepted by Ross and Schroeder should be rejected in light of the counterintuitive consequences it has. In case the disposition involved in belief is overridden, the agent is faced with a choice of whether to accept the believed proposition in her deliberation. In addition, the fact that the agent should not choose to accept the believed proposition does not entail that she lacks justification for believing it. In light of this claim, we offered a detailed description of the kind of higher-order deliberations an agent engages in when she has to determine whether she should accept a given proposition to elucidate the conditions under which an agent is rationally permitted to accept what she believes. Given the way these conditions were clarified, we offered an alternative explanation of the phenomenon of pragmatic encroachment on knowledge which shows how knowledge’s sensitivity to the choice situation an agent is in depends on the agent’s degree of uncertainty for the propositions that she is justified in believing.

## Footnotes

1 The relation between an agent’s credences and her preferences has been formalized in the so-called representation theorems formulated by, e.g., Ramsey (1926), Savage (1954), Jeffrey (1965) and Joyce (1999). They show that the preferences of a rational agent can be represented by a probability function and a utility function which maximize the expected utility of the agent’s possible courses of action.

2 In the context of the present paper, we will use the expressions “treating *p* as true in one’s reasoning”, “leaving ¬*p* out of one’s deliberation” and “evaluating one’s options conditional on *p*’s truth” as synonyms.

3 Some philosophers such as Foley (1992, 2009) and Weatherson (2005) hold that belief and rational belief is definable in terms of high credence and high rational credence. On the other hand, philosophers such as Frankish (2009) and Buchak (2014) argue that such reductive definitions fail to capture fundamental aspects of this doxastic attitude and its conditions of rationality.

4 It is worth noting that Ross and Schroeder’s Reasoning Disposition Account, as they (2014, pp. 268–271) outline, has anti-reductionist consequences regarding the relation between belief and credence.

5 As it will become clear in the next section of this paper, the agent’s confidence that taking into account her uncertainty as to *p*’s truth in the course of her deliberation would change her preferences is itself determined by the stakes involved in the particular choice situation the agent is in. For this reason, although there is a fixed threshold above which an agent’s confidence is sufficient to override her disposition to treat *p* as true, the fact that her disposition is overridden is dependent on the stakes involved in a particular choice situation.

6 Note that it can be irrational for an agent to treat *p* as true in a particular deliberation although her disposition to do so is not overridden. Whether or not it is rationally permissible for an agent to act upon her belief that *p* in a given choice situation is not dependent on the fact that her disposition to treat *p* as true is actually overridden in that choice situation.

7 See also Van Fraasen (1984), Stalnaker (1987), Bratman (1992) and Cresto (2010).

8 This prevents this kind of higher-order decision-problems from generating an infinite regress of the sort discussed by Lin (2014). One first-order decision-problem can lead to many second-order decisions but as each of those second-order decisions is a grand-world decision in Savage’s sense, there is no reason for the agent to engage in third-order deliberations.

9 The agent’s credence in *p* ∗ does not need to result from a conscious process of reasoning to reflect this risk. It is in principle possible that this credence be determined in a more or less automatic manner and reflects this risk.

10 These precisions show to what extent an agent’s credence in *p* ∗ depends on the stakes involved in the choice situation she is in. In addition, they should mitigate the worry that for every first-order decision-problem that an agent faces, there is always vastly many higher-order decision-problems concerning the propositions to accept in the course of her first-order deliberation. While it is true that for every first-order decision-problem, there can be more than one higher-order decision-problem concerning whether or not to accept a proposition, for every proposition *p* relevant to her first-order deliberation, the agent’s credence in *p* ∗ depends on the contextual stakes related to *p*’s truth. As the contextual stakes related to truth of many uncertain propositions are low, many uncertain propositions are treated as true without the need to engage in higher-order deliberations.

11 For a discussion concerning the relation between the cognitive costs of lower-order decisions and the rationality of higher-order decisions, see Maher (1993) and Sargent (2009).
